# Is dUTPase Enzymatic Activity Truly Essential for Viability?

**DOI:** 10.3390/ijms26199260

**Published:** 2025-09-23

**Authors:** Anatoly Glukhov, Ulyana Dzhus, Ilya Kolyadenko, Georgii Selikhanov, Azat Gabdulkhakov

**Affiliations:** 1 Institute of Protein Research RAS, 142290 Pushchino, Russia; gluktol@gmail.com (A.G.); ulya@vega.protres.ru (U.D.); ilya@vega.protres.ru (I.K.); selikhanov@vega.protres.ru (G.S.); 2 Group for Design and Engineering of Industrially Relevant Enzymes and Their Producers, AlmetTech Research Center, ITMO University, 191002 St. Petersburg, Russia

**Keywords:** dUTPase, deoxyuridine triphosphate nucleotidohydrolases, dUTP, protein functions, signaling proteins

## Abstract

The study of protein enzymatic activities has always been a significant area of scientific and industrial research. The key steps typically undertaken in the characterization of a certain enzyme family include establishing the mechanism of catalysis, measuring kinetic parameters, determining structural organization and the architecture of the catalytic center, and subsequent classification. In this review, we tried to touch upon only a few points from the classical description of enzymes of the dUTPase family and added some additional functional properties of a number of representatives of this family. The existence of such extra functions raises questions about the reasons for this function duality. Based on the information known in the literature and our previous research, in this review, we conclude that the enzymatic activity of dUTPases supplements other functions independent of the hydrolysis reaction occurring in the catalytic center. In this context, it seems that dUTP acts not just as a substrate but as a signaling molecule, whose binding induces the realization of a special, non-enzymatic role of dUTPases.

## 1. Introduction

All living organisms use DNA as the storage and carrier of genetic information. Consequently, cells require systems to protect, edit, repair, and accurately transmit this genetic information. One such system is the DNA repair system, which includes members of the deoxyuridine 5′-triphosphate nucleotidohydrolase (dUTPase, Dut) enzyme family. dUTPases are ubiquitous across all domains of life, as well as in numerous eukaryotic viruses and bacteriophages [1,2,3,4,5]. These enzymes catalyze the hydrolysis of dUTP into dUMP and a pyrophosphate residue (PPi) [6]. As a result, on the one hand, the reaction product (dUMP) serves as a precursor for the synthesis of dTTP, a critical component required for DNA replication. On the other hand, this reaction reduces the cellular concentration of dUTP, thereby preventing the erroneous incorporation of uracil into newly synthesized DNA. Although the U:A pair is almost indistinguishable from the canonical T:A pair for DNA-polymerase, the presence of U in newly synthesized DNA triggers the excision repair system [7]. Another way for uracil to appear in DNA is through deamination of cytosine, which leads to mutation. The accumulation of a significant amount of uracil in DNA can lead to cell death due to the generation of numerous double-strand breaks after excision. Therefore, it is critically important to limit the presence of dUTP in the cell, which might otherwise be erroneously utilized by DNA polymerases during DNA replication [6].

The dUTPase family encompasses a large number of enzymes that differ even in their quaternary structures. These enzymes are classified into three groups: homotrimeric, homodimeric and monomeric.

The first group, homotrimers, is the most widespread and the best characterized. Homotrimeric dUTPases are found in nearly all living organisms, as well as in several bacteriophages and eukaryotic viruses (such as adenoviruses, poxviruses, and retroviruses). These enzymes possess three active sites; each formed at the interface of two adjacent subunits, and are characterized by a beta-sheet folding pattern in the polypeptide chain of the monomer [8,9,10,11].

Members of the second group are less studied. They have an alpha-helical folding pattern in the main chain of the monomer [1,12,13,14]. Homodimeric dUTPases have been identified in several parasitic organisms, such as *Leishmania major* [1,15], *Trypanosoma cruzi* [12], *Trypanosoma brucei* [13] and *Campylobacter jejuni* [14] as well as in certain staphylococcal bacteriophages [16].

The least numerous group of dUTPases identified to date consists of monomeric enzymes. These are found in certain eukaryotic viruses, specifically in the *Alpha-* and *Gammaherpesvirinae* subfamilies of the *Herpesvirales* [5,17,18,19,20,21]. Members of this group are characterized by a beta-sheet folding pattern in the main chain and a larger molecular weight compared to members of other dUTPase enzyme families. It is hypothesized that their origin is associated with the duplication of the gene encoding a homotrimeric dUTPase [22,23].

Because dUTPases are widely prevalent in pathogenic organisms, understanding their structural and functional characteristics provides valuable insights for their potential use as new targets in antimicrobial, antiviral, and possibly anticancer therapies [24,25,26,27,28]. For example, dUTPase inhibitors are recognized as effective against infectious diseases such as malaria (caused by *Plasmodium falciparum*) and tuberculosis (caused by *Mycobacterium tuberculosis*), due to the biosynthesis of dTMP in these pathogens is entirely dependent on dUTPase activity. dUTPase is often upregulated in human cancer cells, making it a promising target for anticancer therapy. Studies have shown that the suppression of dUTPase activity in human cells increases their sensitivity to anticancer drugs such as 5-fluorodeoxyuridine, which disrupts dUTP/dTTP levels by inhibiting thymidylate synthase. Therefore, targeting multiple enzymes in the thymidylate metabolism pathway may have synergistic effects in combination therapy. Moreover, it has been demonstrated that the replication of certain viruses in non-dividing (terminally differentiated) cells, where cellular dUTPase activity is low or absent, depends on viral dUTPase. This likely reflects the importance of viral dUTPase in maintaining genomic integrity and highlights its critical role in the replication of these pathogens.

Despite the critical role performed by dUTPases, viable *Escherichia coli* strains encoding an inactive form of dUTPase have been obtained [4]. At the same time, it is believed that these enzymes perform an additional, as-yet unidentified function in the cell. This hypothesis is supported by the observation that dUTPase, along with GroEL, DNA primase, and other *E. coli* proteins, is essential, and knockout of their genes is not feasible [29,30]. Recent studies have also demonstrated that certain dimeric and trimeric dUTPases encoded by bacteriophages regulate the transfer of mobile genetic elements between different strains of *Staphylococcus aureus*, thereby contributing to the dissemination of their pathogenicity genes [2,31,32].

Systematization of information regarding dUTPase structure and functioning and the range of processes in which they may be involved will expand knowledge regarding the importance of dUTPases for cell survival. From the whole pool of available information, we suggest looking at a combination of 4 distinctive features characteristic of this enzyme family: (i) the global prevalence of the enzyme in living cells; (ii) the high structural diversity within the enzyme family; (iii) mentions of involvement in other processes, (iv) the high substrate specificity to only one not very common molecule. According to this context, the following questions arise: Is dUTPase enzymatic activity crucial? Does it constitute the primary function of the enzyme?

## 2. Structural Organization and Enzymatic Activity of dUTPase

As mentioned earlier, all dUTPases are classified into three groups based on their structural organization: homotrimeric, homodimeric, and monomeric. Each group of enzymes is characterized by a distinct folding pattern of the polypeptide chain within the monomer [15,33,34].

**Figure 1:**
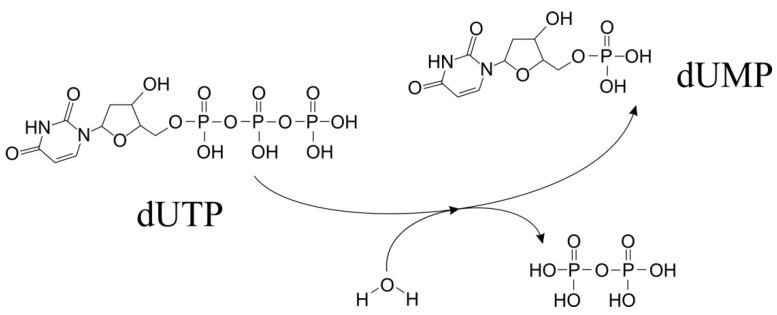
The enzyme reaction (EC3.6.1.23): conversion of dUTP to dUMP, formation and removal of water molecule and pyrophosphate.

**Figure 2:**
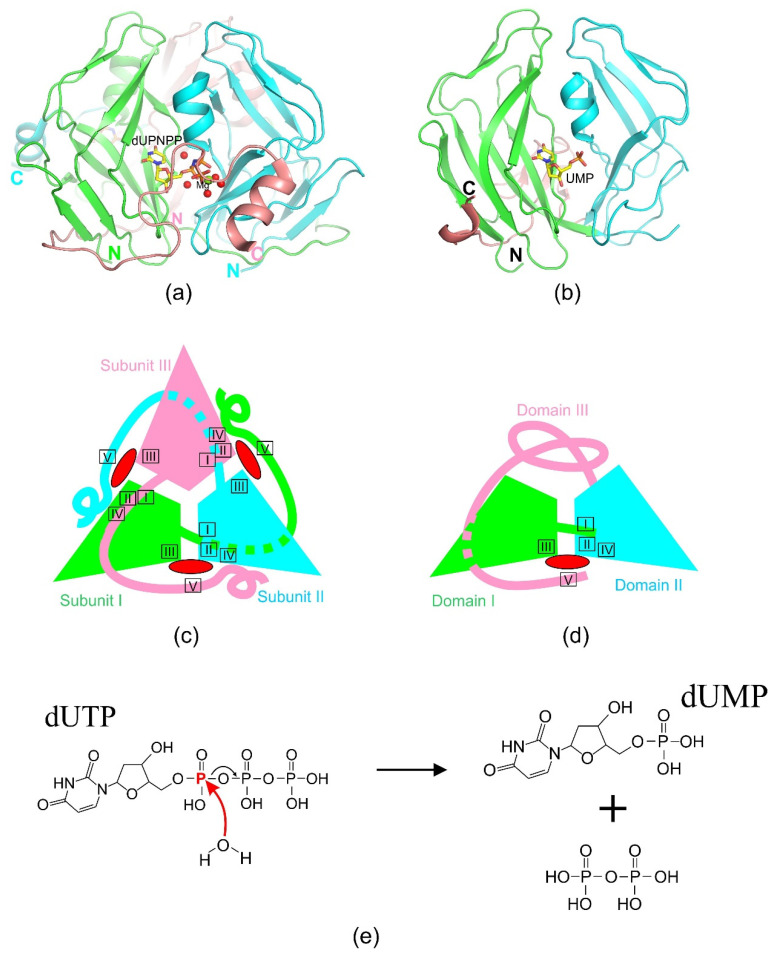
(**a**) View perpendicular to the 3-fold axis of the Swine enzyme looking onto the active site (pdb id 6LJJ); (**b**) View looking onto the active site of the monomeric dUTPases Epstein–Barr Virus (pdb id 2BSY); Schematic organization of the trimeric (**c**) and monomeric (**d**) dUTPases. Localization of the conserved sequence motifs I–V around the active sites represented by red ellipse. (**e**) Reaction scheme showing water nucleophilic attack on gamma-phosphate of dUTP in trimeric dUTPase, leading to the P-O fragmentation to yield dUDP and pyrophosphate.

**Figure 3:**
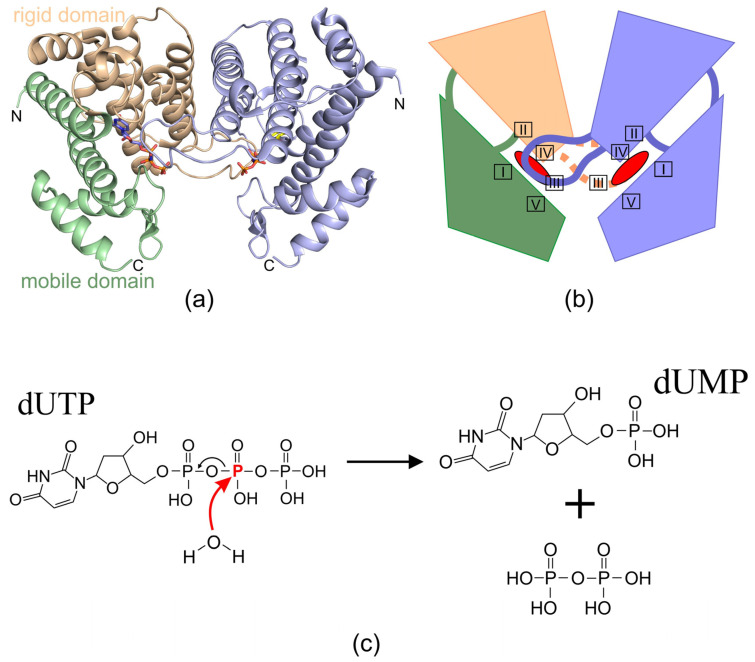
(**a**) View looking onto the active site of the dimeric dUTPase from *Campylobacter jejuni* (pdb id 2CIC); (**b**) Schematic organization of the dimeric dUTPases. (**c**) Reaction scheme showing water nucleophilic attack at beta-phosphate of dUTP in dimeric dUTPase, leading to the P-O fragmentation to yield dUDP and pyrophosphate.

dUTPase enzymes catalyze the hydrolysis of dUTP, producing dUMP and pyrophosphate as reaction products (Figure 1). This reaction has been most thoroughly described for trimeric enzymes [7,35]. The catalytic process follows an S_N_2 mechanism [7]. Despite the principal similarity of reactions of trimeric and dimeric enzymes, a detailed study of the reaction products in the presence of O18- labeled water revealed significant differences in the course of the different enzymes reactions [13,35,36]. In both cases, substrate hydrolysis is initiated by a linear nucleophilic attack of a water molecule Wcat on the phosphate atom, resulting in hydrolysis of the phosphate ester (Figure 2e and Figure 3c). However, in the case of trimeric dUTPases, the nucleophilic attack is carried out towards the alpha-phosphate with O18 remaining in the dUMP molecule, while the nucleophilic attack in dimeric enzymes is carried out on the beta-phosphate, and the radioactively labeled atom ends up in the pyrophosphate [13]. Both similarities and differences in the course of the reaction are reflected in the structural features of the active centers of enzymes from different classes.

Despite the structural variability, the enzymatic activity of all three groups is generally provided by the presence of specific conservative amino acid motifs. The active center is formed by motifs from different subunits or domains and is located between its boundaries.

### 2.1. Homotrimeric dUTPases

This is the most numerous and well-characterized group of dUTPases to date. Its representatives have been identified in plants, animals, fungi, bacteria, various bacteriophages, and certain eukaryotic viruses, such as adenoviruses, poxviruses, and retroviruses [7,8,11,34,37,38]. The three-dimensional structures of a substantial number of trimeric dUTPases encoded by organisms across different kingdoms of life have been determined: *E. coli*, *Homo sapiens*, several bacteriophages, *Arabidopsis thaliana*, and some animal viruses. As implied by the name of this group, the molecule of nearly all homotrimeric dUTPases is composed of three identical polypeptide chains (Figure 2a,c). An exception to this homotrimeric architecture is the dUTPase from *Drosophila virilis*. Its functional enzyme is constituted by a single polypeptide chain that folds into three nearly identical domains. These domains are interconnected by flexible linker sequences and assemble to form a stable, catalytically active structure described as a pseudo-heterotrimer [39,40]. Each polypeptide is structured as an eight-stranded jelly-roll beta-barrel with one or two alpha-helices [8,9]. The amino acid sequence of the monomer contains five conserved motifs evenly distributed along the polypeptide chain. Three active sites are formed by segments from at least two of the three subunits. Specifically, the catalytic cavity is constructed from motif III of one subunit and motifs I, II, and IV of an adjacent subunit. Motif V, located in the C-terminal region, is often difficult to resolve due to its high mobility (Figure 2a,c). However, when visualized, it is observed closing the catalytic cavity upon substrate binding, isolating the reaction and enhancing catalytic efficiency. The stabilization of motif V’s position is mediated by polar interactions between its side-chain residues and the β- and γ-phosphates, as well as hydrophobic interactions between conserved polar residues in the motif and the uracil base.

In most cases, motif V originates from the third subunit, which is not adjacent to the active site. However, exceptions have been reported (e.g., African swine fever virus dUTPase, white spot syndrome virus (wDUT), and *P. falciparum* dUTPase), where the C-terminal region of the protein exhibits a different fold [41,42,43]. In these cases, motif V contributes to the active site architecture in conjunction with motif III from the same subunit. This unique architectural organization, wherein the formation of the active site depends directly on proper trimer assembly, is unusual among enzymes and underscores the structural and functional sophistication of trimeric dUTPases.

For enzymatic function, dUTPase requires a cofactor—a divalent metal ion. Typically, the active site contains a magnesium ion, which is coordinated via water molecules by conserved residues from motifs I, II, and IV. In the case of the conserved aspartate of motif I, coordination is facilitated through two water molecules. The coordinated magnesium ion interacts with the phosphate groups of the triphosphate, stabilizing the conformation of the intermediate (*gauche*) state of the phosphates and thus enhancing hydrolysis efficiency. Upon the reaction completion, magnesium ions may also facilitate the cooperative evacuation of the pyrophosphate product from the active site. This is supported by the fact that certain crystal structures of dUTPases, in their apo-form or in complex with dUMP, do not contain magnesium ions in their active site [41,44]. At the same time, Mustafi D. et al. demonstrated a new role of divalent metal ions in catalysis [45]. The authors note that dUTPases can cleave the substrate even in the absence of a cofactor, and the presence of a divalent metal ion increases the catalytic activity of the enzyme.

Specificity for uracil and exclusion of purines and thymine are ensured by the β-hairpin formed by conserved residues of motif III [9,46]. In this process, positioning of uracil via hydrogen bonds involves atoms of the main chain rather than side chains, emphasizing that the folding of this region is more critical than the side chain composition. The same hairpin also provides selectivity for deoxyribose, with the conserved tyrosine preventing steric hindrance that would allow ribonucleotide binding. Selectivity for the triphosphate backbone is mediated by conserved residues from motifs I, II, IV and the phosphate conformation induced by the magnesium ion. The spatial structures of dUTPase complexes, where the dUDP is present, show the nucleotide in a ‘non-active’ trans-state of the phosphate backbone. In spite of high specificity of trimeric dUTPases, some of them have been predicted (calculated) to bind dATP and dCTP without hydrolytic activity [47].

A unique member of this group of enzymes is Dut of *D. virilis* [39,40]. Unlike all other homotrimeric dUTPases, this enzyme consists of only one polypeptide chain composed of three nearly identical (87.76% identity) repeating amino acid sequences separated by linker peptides. *D. virilis* dUTPase also has three active sites per molecule, and each example of the pseudoheterotrimer, same as an individual subunit of the homotrimer, contains five characteristic conserved motifs and, apparently, a jelly-roll beta-barrel fold. It is suggested that the origin of this form comes from duplication and fusion of the dut gene, as in the case of monomeric Dut Epstein–Barr virus [44] and pseudorabies virus [33]. But for the last ones, the numerous mutations led to only one set of conservative motifs, and one active center was retained.

### 2.2. Homodimeric dUTPases

This is a small group of dUTPases. They have been identified in several pathogenic microorganisms as well as in certain bacteriophages that infect *Staphylococcus aureus* cells [32,48,49]. In contrast to the previous group, to date, there are only four representatives of homodimeric dUTPases that have crystallographic structures available. The three-dimensional structures have been determined for enzymes from pathogenic organisms such as *T. cruzi* (pdb id 1OGL), *L. major* (pdb id 2YAY), and *C. jejuni* (pdb id 2CIC), as well as for the dUTPase of the staphylococcal phage ϕD1 (pdb id 5MYF).

A characteristic feature of dimeric dUTPases is that their monomers are composed of a large number of interwoven α-helices connected by flexible loops [1,12,14,16]. For example, the dUTPases from *L. major* (LjdUTPase) [50] and phage ϕD1 consist exclusively of α-helices (10 and 8 α-helices, respectively), while the dUTPases from *C. jejuni* (CjdUTPase) [51] and *T. cruzi* (TcdUTPase) [52] comprise 11 and 12 α-helices, respectively, along with 2 short β-sheets. Each monomer of a dimeric dUTPase consists of two domains. One domain, the “rigid” domain, participates in the dimerization of subunits and forms the enzyme core. The other, the “flexible” domain, consists of two subdomains formed by the N- and C-terminal amino acid sequences of the monomer, respectively (Figure 3a,b). This domain is positioned at an angle relative to the “rigid” domain, and the entry of the substrate into the enzyme’s active site induces a shift in its position by up to 20 Å. Moreover, significant rearrangements of secondary structure elements occur within the “flexible” domain. For instance, structural changes are observed in helix h9 of TcdUTPase, while helices h10 and h12 split into two smaller helices each. Additionally, two new short β-strands are formed. These transformations alter the relative positions of the secondary structures in the “flexible” domain, leading to the formation of a more stable and compact conformation [12].

The polypeptide chains of homodimeric dUTPases, like those of homotrimeric enzymes, contain five conserved motifs evenly distributed along the entire sequence (Figure 3b). However, the architecture of their active sites share no similarity with those found in trimeric enzymes [1,12,13,14,15,16]. Two active sites are symmetrically located at the interface of the dimeric dUTPase molecule, with each substrate-binding pocket positioned in a groove formed between the “rigid” and “flexible” domains of a single monomer. As a result, the catalytic pocket of homodimeric dUTPases is composed of conserved motifs I, II, IV, and V from one protomer, located in the α-helical regions of the “rigid” and “flexible” domains. A long loop, often referred to as the “latch”, extends between helices in the inter-subunit space and reaches the neighboring subunit, providing its motif III to contribute to the formation of that subunit’s active site (Figure 3b).

Unlike trimeric dUTPases, where dUDP acts as an inhibitor [36], dimeric enzymes are capable of hydrolyzing both dUTP and dUDP. For this reason, it is suggested that the reaction mechanism may differ from that of trimeric enzymes, requiring more detailed investigation (Figure 3c). Dimeric dUTPases are inhibited by dUMP [15]. In addition, these enzymes exhibit reduced nucleotide specificity, demonstrating the ability to additionally hydrolyze dCTP. Such decreased specificity may indicate that the active site of dimeric dUTPases has a less rigid organization, allowing the process to occur with greater variability.

The active site of dimeric dUTPases contains either three or two magnesium ions, depending on the ligand present within the active site. Two of these magnesium ions coordinate the catalytic water molecule. In general, amino acid residues from motif I participate in the coordination of the U/C base. Magnesium ion coordination is mediated by residues from motifs II and IV, with motif IV also being responsible for deoxyribose selection. Motif V, located within the flexible domain, together with motif III from the neighboring subunit, coordinates the positioning of the phosphate groups.

It is worth noting that the analysis of amino acid sequences (X-ray structural and bioinformatic analyses) of homodimeric dUTPases, as well as members of the protein families HisE, MazG, and dCTPase of enterophages T2 and T4, suggests that they all belong to a single superfamily of all-α NTP-pyrophosphotase [48,53,54]. Members of this superfamily are characterized by a shared polypeptide chain folding pattern and similar catalytic properties and functions, specifically in scavenging abnormal NTPs that arise during cellular metabolism. The authors propose that the MazG-like subunit represents a common ancestor in the evolutionary history of this superfamily of enzymes. For example, it is hypothesized that homodimeric dUTPases originated (or evolved) through duplication, fusion, and the subsequent loss of one catalytic center in the C-terminal MazG-like domain.

### 2.3. Monomeric dUTPases

Monomeric dUTPases are found exclusively in viruses of the *Herpesviridae* family, members of which infect mammals, birds, and reptiles. Genes encoding monomeric enzymes have been identified in herpes simplex virus (HSV-1) [20], varicella-zoster virus (VZV) [22], Epstein–Barr virus (EBV) [44], and pseudorabies virus (PRV) [33], belonging to the subfamilies *Alphaherpesvirinae* and *Gammaherpesvirinae*.

Currently, three-dimensional structures are available only for two representatives of monomeric dUTPases. The first structure of the Epstein–Barr virus Dut was determined in 2005 (pdb id 2BSY) [44], and nearly two decades later, the structure of the pseudorabies virus dUTPase was solved (pdb id 8ZWQ) [33]. These are two-domain proteins, approximately twice the size of a monomer of cellular homotrimeric analogs. However, comparative analyses of amino acid sequences and 3D structures of mono- and trimeric dUTPases reveal significant similarities [33,44]. For example, similar to homotrimeric dUTPases, enzymes in this group exhibit an almost entirely β-strand structure with a few (1–3) short α-helices. The amino acid sequences of monomeric Dut enzymes also contain five conserved motifs characteristic of homotrimeric dUTPases, albeit arranged differently along the polypeptide chain. The shift of motif III toward the N-terminus of the protein results in the following sequence: motifs III, I, II, IV, and V. A single active site is located between rigid and mobile domains (Figure 2b,d). It is organized by four out of the five classical motifs described for trimeric dUTPases. Motif V is present in the active site sequence but is not visualized in the described structure due to the high mobility of the C-terminus. All two domains contain nearly the full complement of the five conserved motifs characteristic of trimeric dUTPases, with some exceptions. The catalytic center is formed by motif III from the first domain and motifs I, II, and IV from the second domain, which surround a cavity created by the two domains. Motif V, located in the mobile C-terminal region of the protein as in homotrimeric dUTPases, likely plays a catalytic role. The motifs forming the single active site exhibit high conservativeness, while those located on other domain surfaces are variable or may be absent. Substrate binding within the active site results in the formation of a similar hydrogen bond network and magnesium ion coordination as observed in trimeric enzymes [33,44]. Overall, it can be confidently stated that the reaction mechanism in the active site of the EBV dUTPase is analogous to that of the trimeric enzymes. Despite the low sequence identity between domains I and II of monomeric dUTPases and the subunits of homotrimeric enzymes, all exhibit a similar jelly-roll beta-barrel fold. Consequently, the two domains of a monomeric dUTPase function analogously to the two subunits of homotrimeric Dut enzymes in forming a single active site.

The origin of monomeric forms of dUTPases is associated with the duplication of an ancestor and subsequent fusion of two copies of the *dut* gene [5,23]. Given that monomeric dUTPase has thus far been identified only in eukaryotic viruses, it is hypothesized that an intensive subsequent mutation process led to the loss of several conserved motifs. This resulted in a distinct arrangement of motifs along the polypeptide chain, while the characteristic jelly-roll β-barrel structural fold was retained.

A unique representative of this group of enzymes is the pseudorabies virus (PRV), a member of the *Alphaherpesvirinae* subfamily within the swine *Herpesviridae* family. As a dimeric dUTPase, its sequence demonstrates higher homology to the active site sequences of herpesvirus enzymes than to those of described dimeric bacterial enzymes [33]. Alignment reveals six homologous motifs, with conserved residues in five of them contributing to the formation of the active site [33]. Notably, homology to the Epstein–Barr virus (EBV) dUTPase is also observed at the architectural level within the active site, despite the fact that the EBV dUTPase is a monomer. In this context, it would be reasonable to classify this enzyme either within the monomeric group or as a separate group of enzymes specific to the *Herpesviridae* family. Additionally, it is worth noting that disruption of the dimeric structure leads to a significant decrease in enzymatic activity. It is also worth noting that the structure of the active sites of the monomers within a single PRV dUTPase molecule differs in topology. One of the monomers exhibits the formation of an additional short α-helix, which undoubtedly contributes to differences in enzymatic activity [33].

Interestingly, PRV Dut exists in a dimeric form in both crystal state and solution [33]. However, unlike homodimeric dUTPases, dimerization of PRV Dut is mediated by a network of hydrogen bonds and hydrophobic interactions between four β-sheets: the short regions P33-V36 and R242-A248 in the N- and C-terminal regions of the protein, respectively. This dimerization stabilizes the protein, which in turn impacts its enzymatic activity. Nevertheless, it cannot be excluded that the oligomeric state of PRV Dut enables additional functional roles during viral replication [33].

## 3. dUTPase Functions in Living Organisms

The importance and necessity of dUTPase enzymatic activity for living organisms are, at first glance, indisputable. Indeed, Duts are integral components of the cellular system responsible for maintaining the integrity of genetic material.

Carrying out the hydrolysis of dUTP to dUMP and pyrophosphate the enzyme has a strong influence on two critical cell process. First, it maintains a balanced dUTP/dTTP ratio within the cell, thereby preventing the incorporation of uracil into newly synthesized DNA. Excessive uracil incorporation during replication can result in numerous irreparable double-strand breaks, ultimately leading to cell death. Second, the reaction product, dUMP, is a key precursor for dTTP synthesis. In pathogenic organisms such as *P. falciparum* and *M. tuberculosis*, which cause malaria and tuberculosis, respectively, dUMP is the sole precursor for dTTP biosynthesis in the cell [42,55].

At the same time, bioinformatic analysis of bacterial and archaeal genomes has revealed amazing fact that the absence of the *dut* gene is not an exceptionally rare phenomenon among the genomes analyzed [6]. For instance, 14 out of 15 genomes studied within the *Thermotogae* phylum lack *dut* genes. The same absence of *dut* genes has also been demonstrated in representatives of the *Planctomycetes*, *Tenericutes*, *Firmicutes*, *Cyanobacteria*, *Spirochaetes*, *Bacteroidetes*, and *Euryarchaeota*. However, it should not be justly ruled out that the dUTP hydrolysis function in these organisms may be performed by other, as yet unidentified, proteins, such as those from the MazG protein family.

From all the known data, it deserves special emphasis that a significant amount of experimental evidence has accumulated in the literature, highlighting an importance of the mere presence of the dUTPase in the cell rather than its enzymatic function. For example, it has been previously mentioned that the *E. coli* dUTPase belongs to a group of proteins for which gene knockout is not feasible [4,29,30]. However, *E. coli* strains encoding inactive dUTPase do not exhibit significant phenotypic changes. Similar results, highlighting the importance of dUTPase, have also been reported for *Saccharomyces cerevisiae* [3]. In this regard, the idea that dUTPases may possess an additional, as-yet unknown critical function that appears to be species- or genus-specific is gaining increasing popularity in the literature. The following experimental data may serve to support this hypothesis.

The *Mycobacterium smegmatis* dUTPase contains a short additional motif VI, consisting of only five amino acid residues, adjacent to motif V in the flexible C-terminal region of the protein. As in the case of *E. coli*, the knockout of the *dut* gene in *M. smegmatis* is lethal for the organism. Although the deletion of this motif does not affect the enzymatic activity of the protein, such mutant form is unable to restore the wild-type phenotype [56]. It is noteworthy that the identified additional motif VI appears to be genus-specific: it has also been identified in the dUTPases of pathogenic organisms such as *M. tuberculosis* and *M. leprae*, where it may also play a role in critical intracellular processes [55,57].

The dUTPase of *Drosophila melanogaster* is notable for existing in two isoforms in actively dividing cells [58,59]. These isoforms, nuclear and cytoplasmic, are generated through alternative splicing and differ by a short nuclear localization signal sequence located at the N-terminal region of the protein. The primary function of the “nuclear” dUTPase is well understood, whereas the function of the “cytoplasmic” isoform remains to be determined. One possible explanation is that this isoform serves an additional role in a signaling pathway.

It is worth noting that *D. melanogaster* dUTPase also contains a species-specific additional sequence of 28 amino acid residues in the C-terminal region of the protein. A similar situation has been observed with rat dUTPase, which has been shown to play a regulatory role in signaling by inhibiting peroxisome proliferator-activated nuclear receptor alpha [60]. The additional sequence in the N-terminal region of the protein is assumed to play an important role in this function. Taken together, these findings support the idea that dUTPases in eukaryotic organisms, like their prokaryotic counterparts, may perform additional functions within cells.

Equally significant and intriguing findings have been obtained from studies of viral dUTPases. Among bacterial viruses, the most extensively studied phenomenon is the induction of SaPI (*Staphylococcus aureus* Pathogenicity Island) mobilization through the interaction of phage-encoded dUTPase with the Stl repressor [2,10,31]. SaPIs are genetic elements capable of mobilizing and disseminating virulence genes among *S. aureus* strains [61,62,63]. Under the regulation of Stl, a global repressor encoded by SaPI itself, these genetic elements remain dormant within the host chromosome. However, upon infection of *S. aureus* cells by a bacteriophage, SaPIs are excised, undergo autonomous replication, and are packaged into phage-like particles composed of virion proteins [64,65]. This process results in an exceptionally high frequency of both inter- and intraspecies transfers [66,67]. Both trimeric and dimeric forms of phage-encoded Dut proteins act as antirepressor proteins for specific SaPIs, such as SaPIbov1, SaPIbov5, or SaPIov1 [2,31,32,68]. Comparative analysis of amino acid sequences of homotrimeric Duts from various staphylococcal phages has revealed significant sequence similarity, except for a non-conserved central region designated as motif VI [2]. This motif is absent in some phage-encoded Duts. At the same time, mutant forms of dUTPases lacking motif VI retain enzymatic activity either completely lose the ability to induce SaPI mobilization, as in the case of Dut phage 80α, or significantly increase the efficiency of the function (φ11 Dut) [10]. The binding of the Stl repressor by phage dUTPases, while independent of the enzymatic activity of the protein, is competitively influenced by the presence of dUTP in the environment.

Similar findings have been reported for dUTPases of T5-like bacteriophages [8]. It has been demonstrated that the homotrimeric dUTPase of phage T5 performs an additional function during the lytic cycle of the virus, and knockout of its gene leads to dramatic consequences, disrupting phage development within host cells. This additional function, as with other dUTPases, is independent of the enzymatic activity of the protein but is strongly influenced by the presence of a short, additional sequence in the N-terminal region of the protein.

A substantial body of evidence also highlights additional functions of dUTPases encoded by viruses of eukaryotic organisms. For instance, some eukaryotic virus-encoded Duts can modulate the immune response of the infected host. For example, the dUTPase encoded by the pseudorabies virus (PRV) induces lysosomal degradation of the type I interferon receptor, thereby suppressing the alpha-interferon response [69]. Similarly, the dUTPase of MHV-68, while not essential for viral replication, inhibits the expression of the type I interferon signaling pathway [70]. Moreover, the Dut protein from the Kaposi’s sarcoma-associated herpesvirus (KSHV) has been reported to suppress the immune response by targeting multiple cytokine receptors. Interestingly, this immunosuppressive function is also independent of the enzymatic activity of the protein [71]. These findings suggest that viral Duts have developed unique immunoregulatory functions.

## 4. Conclusions

Descriptions of representatives from the class of deoxyuridine triphosphate nucleotidohydrolases traditionally begin with an overview of their enzymatic activity and their capacity to regulate the dUTP/dTTP balance in the organism or host cells. It is invariably noted that a deficiency of this enzyme severely compromises survival, with gene knockouts being lethal. Such outcomes are often bluntly attributed to the accumulation of mutations in actively replicating DNA and the overload of DNA repair systems. However, several observations challenge this plain concept.

First, while the knockout of the *dut* gene is lethal, mutants unable to hydrolyze dUTP are frequently viable. Second, some species lack *dut* genes altogether. Third, it has been demonstrated that the frequency of uracil misincorporation during replication, though elevated (approximately six times the standard level), is not high enough to account for total cellular death [6]. Moreover, proteins other than dUTPases can hydrolyze dUTP and may potentially compensate for the absence of the *dut* gene.

At the same time, a growing body of research highlights the involvement of dUTPases in processes beyond their enzymatic activity, some of which are unrelated to DNA replication or the dUTP/dUMP balance. Furthermore, numerous examples now exist where the elimination of enzymatic activity—i.e., the ability to hydrolyze dUTP into dUMP and pyrophosphate—does not affect certain parallel properties or functions of dUTPases.

Combining these observations suggests a novel perspective on dUTPases as a group of proteins that are functionally diverse and independently engaged in various vital cellular cascades while retaining canonical dUTPase activity in either a trimeric or dimeric structural architecture. Consequently, when describing new representatives or functions of dUTPases, it is essential to go beyond enzymatic homology analyses. If one accepts the idea that dUTPase activity coexists with other functions, the high variability in amino acid sequences outside catalytic motifs becomes understandable. Moreover, given that the absence of dUTP hydrolysis itself does not cause critical harm to the organism, unlike the knockout of the *dut* gene, it is likely that the presence of the enzyme itself is crucial, and even the ability to bind dUTP may be sufficient. Therefore, studies should focus on mutations that prevent dUTP binding rather than merely inhibiting hydrolytic capacity. An example of such an approach is the A73L mutation in the dUTPase of bacteriophage ϕDI, which prevents dUTP binding and serves as a model for understanding functional divergence in dUTPases [13].

In light of this concept, the perception of dUTP as a substrate requiring processing undergoes a shift. From being merely a nucleotide—a building block of DNA or an auxiliary supplier of dTTP precursors—it transitions to the status of a molecule used for additional alternative purposes. A comparable example can be found in the roles of GTP and ATP nucleotides. The classes of GTPases and ATPases encompass a wide variety of proteins that utilize the energy derived from GTP or ATP hydrolysis to perform their primary functions (here, we do not take into account G-proteins but keep in mind, for example, transcription and translation factors and enzymes). Structurally, these proteins share domains for binding their respective nucleotides, but their primary functions can differ significantly. In this context, the dUTP molecule may also be meant as an effector whose binding and/or hydrolysis is essential for executing another protein function. Here, “signal transmission” could be mediated by structural rearrangements upon dUTP binding, such as increased mobility in the C-terminal region of trimeric dUTPases or the transition of two-domain enzymes from an open to a closed form.

Thus, it can be hypothesized that, unlike the energy-providing GTP and ATP, dUTP primarily acts as an effector or trigger in fine-tuned regulatory systems. It is no coincidence that *dut* genes are found across all domains of life, including certain viruses. In viruses—organisms where rapid responses to environmental changes and adaptation of life-sustaining systems are particularly critical—unconventional variants of dUTPase are often observed. These variants, although enzymatically active, frequently exhibit species-specific conservativity outside of catalytic motifs. This fact has led researchers to propose viral dUTPase as a promising target for pharmaceutical development.

The notion that the functional diversity of dUTPases arises from a variety of additional functions is supported by cases of discrepancy in the classification of certain enzymes. Traditionally, dUTPases are categorized based on their structural organization, both at the protein level and within the active site. However, there are instances where grouping certain enzymes might better reflect their alternative functions. For example, among staphylococcal viral dUTPases, both trimeric and dimeric representatives exist. Nonetheless, the majority share the ability to interact with the Stl repressor and trigger the mobilization of pathogenicity islands via a common mechanism.

In our studies, dUTPases of T5-like bacteriophages exhibit a distinctive feature: a small loop at the apices of the trimer [8]. This loop demonstrates species-specific conservation within the family, despite variability in its length and sequence. Another example is the dUTPase of PRV. Despite being a dimer with a single active site located between two domains, as is typical for dimeric enzymes, PRV dUTPase might be more appropriately classified as a monomeric enzyme. Furthermore, PRV exhibits phylogenetic affinity to trimeric herpesvirus dUTPases.

At present, the classical classification remains the only feasible approach, as identifying additional functions in dUTPases is a challenging task. An even greater challenge lies in pinpointing specific features associated with these additional functions based solely on amino acid sequences. However, as a sufficient pool of knowledge accumulates in this field, we may eventually transition to a more refined classification system for this protein family.
